# Algal Feedback and Removal Efficiency in a Sequencing Batch Reactor Algae Process (SBAR) to Treat the Antibiotic Cefradine

**DOI:** 10.1371/journal.pone.0133273

**Published:** 2015-07-15

**Authors:** Jianqiu Chen, Fengzhu Zheng, Ruixin Guo

**Affiliations:** Department of Environmental Science, China Pharmaceutical University, 210009, Nanjing, China; NIEHS/NIH, UNITED STATES

## Abstract

Many previous studies focused on the removal capability for contaminants when the algae grown in an unexposed, unpolluted environment and ignored whether the feedback of algae to the toxic stress influenced the removal capability in a subsequent treatment batch. The present research investigated and compared algal feedback and removal efficiency in a sequencing batch reactor algae process (SBAR) to remove cefradine. Three varied pollution load conditions (10, 30 and 60 mg/L) were considered. Compared with the algal characteristics in the first treatment batch at 10 and 30 mg/L, higher algal growth inhibition rates were observed in the second treatment batch (11.23% to 20.81%). In contrast, algae produced more photosynthetic pigments in response to cefradine in the second treatment batch. A better removal efficiency (76.02%) was obtained during 96 h when the alga treated the antibiotic at 60 mg/L in the first treatment batch and at 30 mg/L in the second treatment batch. Additionally, the removal rate per unit algal density was also improved when the alga treated the antibiotic at 30 or 60 mg/L in the first treatment batch, respectively and at 30 mg/L in the second treatment batch. Our result indicated that the green algae were also able to adapt to varied pollution loads in different treatment batches.

## Introduction

In recent years, antibiotics have been widely used in the field of human and veterinary medicine, as well as in aquaculture [1]. With increasing antibiotics use, however, only a very small proportion of the medication can be absorbed by the organism [2]. In addition, a large portion of the antibiotic is excreted into the environment by a variety of routes [3]. In general, antibiotics for human therapy and their metabolites are discharged into municipal wastewater and then reach the sewage treatment plant. The antibiotics that are not completely removed by the sewage treatment processes will directly reach surface water [4]. Cases in which antibiotics acted as a source of organic contaminants in surface water have been reported since 1982 [5–8]. Many antibiotics in the environment could be eliminated in a relatively short time; nonetheless, they are also regarded as highly persistent pollutants because of their continuous infusion into the environment [9]. Antibiotics in the environment may induce antibiotic-resistant bacteria and antibiotic-resistantce genes [10–12] and result toxic effects on aquatic species [13,14]. Therefore, an integrated risk assessment of antibiotics for human health and the environment should be performed.

Microalgae are a primary producer in food webs, and detrimental effects on these organisms could elicit subtle but significant effects on the entire food chain [15]. Studies have demonstrated that microalgae have the capability to accumulate and remove environmental contaminants, such as heavy metals, insecticides and other chemicals. There are several applications of algae for the removal of Cd (II), Pb (II) [16], Cr [17], bisphenol A [18], fluroxypyr (pesticide) and tetracycline (herbicide) [19,20]. In addition, the green algae *C*. *pyrenoidosa* helped with the removal of tetracycline during a wastewater biological treatment [21]. *Microcystis aeruginosa* was able to degrade 12.5%-32.9% of spiramycin and 30.5%-33.6% of amoxicillin [22]. Cephalosporins, which are broad-spectrum antibiotics, have been commonly applied in humans, animals and aquaculture, and account for approximately 60.0% of the total antibiotic consumption [23]. The compounds had not undergone measurable biodegradation in the natural aquatic environment [24] and only 3–10% could be biodegraded by the traditional treatment process in urban wastewater treatment plants (UWTPs) [25]. In our previous study, excellent removal efficiency of cephalosporins has been achieved by an algae-activated sludge combined system [26].

All of these results indicated that algae has a promising and efficient degradation capacity on contaminants. Most of the studies, however, focused on the removal capability of algae, which grown in an unpolluted environment before the treatment and ignored whether the feedback of alga to the toxic stress influenced the removal capability in a subsequent treatment batch. Algal tolerance of contaminants plays a decisive role in continuous pollution treatment processes. It is possible that the sensitivity or tolerance of algae changes after the first treatment and therefore causes feedbacks during continuous treatment that influences the final removal efficiency. For example, although 57.0% of fluroxypyr and 66.0% of prometryne were degraded by green alga during a 5-day treatment [19, 20], or a very high removal rate (99%) of bisphenol A was obtained during a 16-day treatment [18]; whether the algae in the continuous treatment process maintained the same removal capability throughout the treatment is not known. Thus, evaluation of treatment efficiency could focus on more than the first treatment. The feedback of the algae would also influence the removal capability in subsequent treatment batches. The aim of this research is to investigate and compare algal characteristics and the removal efficiency in a sequencing batch reactor algae process (SBAR) to remove a widely used cephalosporin, cefradine. Varied concentrations of the antibiotic in the input water were also considered.

## Materials and Methods

There was no human or vertebrate animal subjects and/or tissue used in the present study. The only used organism was *Chlorella pyrenoidosa*, a freshwater alga species which was obtained from the Institute of Hydrobiology of the Chinese Academy of Sciences.

### Antibiotics and analysis procedures

The antibiotic cefradine used in the present work was purchased from Yabang investment holding group Co., Ltd. The reaction, that is the removal of the antibiotic by the green alga, was evaluated. The actual concentrations of the antibiotic were determined by HPLC. Cefradine was separated and determined with an Inertsil ODS column (4.6 mm × 150 mm, 5 μm). The mobile phase to analysis the antibiotic as follows: water-methanol-3.86% sodium acetate-4% acetic acid (682:300:15:3). The flow rate was 1.0 mL/min at ambient temperature. All detections were performed by UV absorption with a wavelength of 254 nm.

### Algal treatment

The algal cells were incubated with BG-11 medium and maintained at 25 ± 1°C under an illumination intensity of 2000 lux, with a 12 h/12 h light/dark cycle. *C*. *pyrenoidosa* was pre-cultivated under the optimized condition for at least two weeks before the treatment. The initial concentration of algae was 20 × 10^7^ cells/mL. Density measures were repeated three times at the given time during treatment.

To compare algal characteristics and removal efficiency in different parts of the treatment, evaluations were performed in two parts: the removal rate of cefradine and the characteristics of the green algae were determined in the first treatment batch and then in the second treatment. In the first treatment batch, the corresponding input concentrations of the antibiotic were set at 10 mg/L (i.e., low pollution load), 30 mg/L (i.e., moderate pollution load) and 60 mg/L (i.e., high pollution load). The hydraulic retention time (HRT) was set at 48 h. Residual cefradine in the treatment system was determined at 24.0 and 48.0 h. The values were used to calculate the removal rate. Moreover, the algal growth capacity and changes in chlorophyll-a were also evaluated during the treatment at the given time. The cells were observed microscopically. Algal chlorophyll was extracted using 80% acetone. The sample was centrifuged at 4000 rpm for 10 minutes. Next, it was placed in a spectrophotometer to measure the light absorbance [27]. Additionally, three relationships have been considered and classified between the first and second treatment batches (see Table 1). All treatment batches were maintained at the same temperature (25 ± 1°C) and illumination intensity (2000 lux) as that in the algal pre-culture with a 12 h/12 h light/dark cycle.

**Table 1:** The concentration of cefradine in the first and second treatment batches.

Treatment Group	First treatment batch (mg/L)	Second treatment batch (mg/L)
**1.1**	10	30
**1.2**	10	60
**2.1**	30	30
**2.2**	30	60
**3.1**	60	30
**3.2**	60	60
**Un-treatment**	0	0

Treatment groups 1.1, 1.2 and 2.2 simulated conditions with a low or moderate pollution load in the first batch and an increased pollution load in the second batch, respectively. Treatment groups 2.1 and 3.2 simulated a constant pollution load across the first and second batches, while treatment group 3.1 simulated a condition, in which the pollution load was decreased after the first batch. Considering the impact of the antibiotic on the subsequent algal treatment batch, the HRT in the second treatment batch was extended to 96 h. The residual cefradine, algal growth capacity and change in chlorophyll were determined at 24.0, 48.0 72.0 and 96.0 h, with the same methods. Algae with no treatment were used as a control. Each treatment group were run 3 parallels.

### Statistical analyses

All data analyses were carried out with SPSS analytic package 16.0. Data were first tested for homogeneity (Levene’s test). Variables from the results of every treatment group were examined by one-way analysis of variance (ANOVA) to identify significant differences. All figures were produced using Sigmaplot version 12.0.

## Results and Discussion

**Fig 1:**
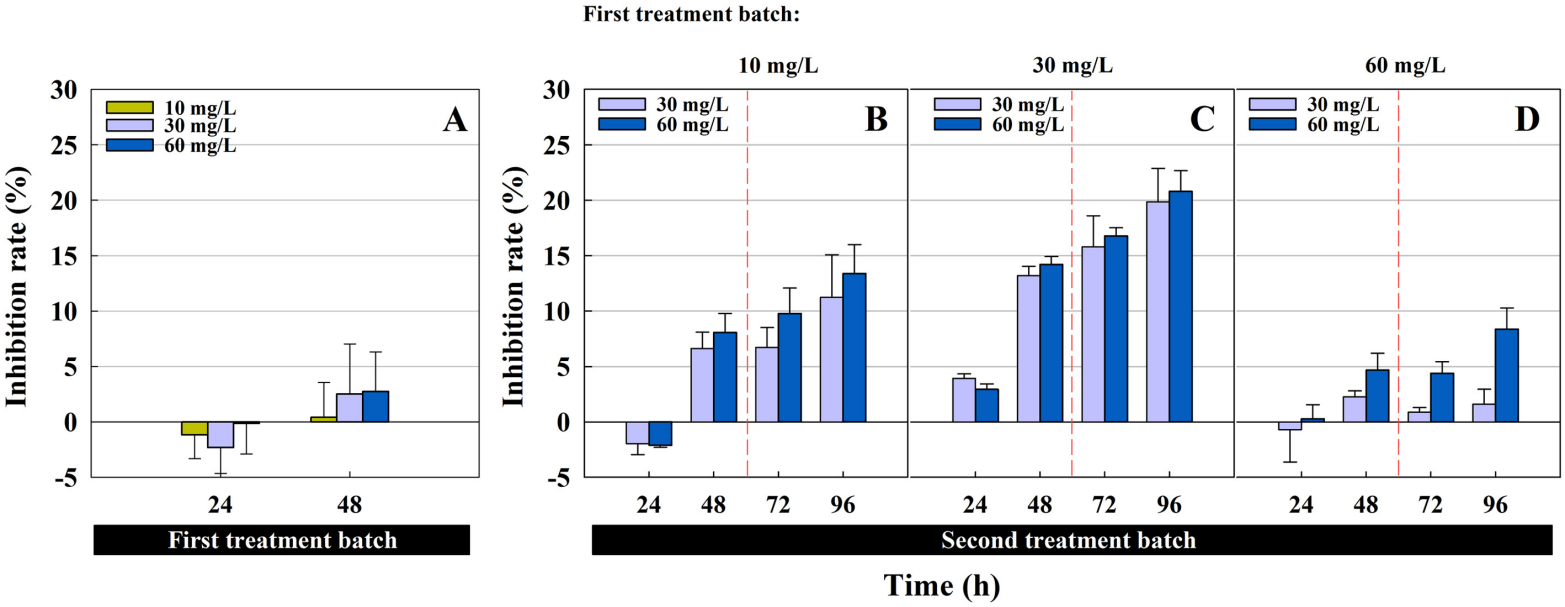
The population growth inhibition rate of *C*. *pyrenoidosa* in a sequencing batch reactor alga process (SBAR) to remove cefradine. (A) The inhibition rate in the first treatment batch at three concentrations (10, 30 and 60 mg/L). (B-D): The inhibition rate in the second treatment batch at two concentrations (30 and 60 mg/L) after a first treatment batch at 10 mg/L, 30 mg/L or 60 mg/L.

The inhibition rate of *Chlorella pyrenoidosa* in the first and second treatment batches is shown in Fig 1. The inhibition rate was -1.17 ± 2.14%, -2.30 ± 2.33% and -0.11 ± 2.78% after 24 h under the first treatment batch of cefradine at 10, 30 and 60 mg/L, respectively. The results indicated that the population of green algae increased slightly under cefradine during the first 24 h. However, the antibiotic inhibited algal growth significantly at 48 h (*p*<0.05), and the impact became more sever with increasing concentrations. The response of the algae to the second treatment batch at the given concentrations has also been evaluated. Following a first treatment with the lowest concentration (i.e., 10 mg/L), the inhibition rates of the green algae under a second treatment batch at 30 and 60 mg/L are presented in Fig 1B. Compared to the no treatment group, the algae in two groups (i.e., groups 1.1 and 1.2) under the given concentration of cefradine exhibited better growth during the first 24 h. However, the inhibition rates increased rapidly up to 6.63 ± 1.47% and 8.07 ± 1.71% at 48 h and then increased continuously up to 96 h. Our results also indicated that the impact occurred in a concentration-dependent manner; a higher inhibition was observed at 96 h in algae that were exposed to 60 mg/L in the second treatment batch than those were exposed to 30 mg/L. In addition, after a first treatment batch at 30 mg/L, growth inhibition of the algae (i.e., group 2.1 and 2.2) was observed at the beginning of 24 h regardless of whether the algae were exposed to the antibiotic again at strengths of 30 or 60 mg/L (Fig 1C). As time went on, the inhibition rate also increased to 19.85 ± 3.03% and 20.81 ± 1.86% at 96 h, (i.e., at 30 and 60 mg/L, respectively). This result was consistent with the observation that the algal suspensions were yellow at the end of the treatment; while this was not observed in the no treatment group. These results suggest that the impact of cefradine on green alga occurred gradually and could inhibit algal growth in a long time. Finally, as shown in Fig 1D, the inhibition rate fluctuated from -0.70 ± 2.91% to 2.27 ± 0.52%, then declined to 0.88 ± 0.41% and finally researched 1.59 ± 1.36% over the course of 96 h in algae exposed to 30 mg/L of antibiotic in the second treatment batch. There was a similar change when *C*. *pyrenoidosa* was used to treat the antibiotic at 60 mg/L; in this case, the rate changed from 0.28 ± 1.28% to 4.66 ± 1.54%; then fell to 4.39 ± 1.06% and finally climbed to 8.37 ± 1.92%. The maximum inhibitory rates obtained in Fig 1D were 2.27 ± 0.52% and 8.37 ± 1.92% at 30 and 60 mg/L, respectively. It is worth noting that the values in groups 3.1 and 3.2 were lower than those in other groups, which treated the antibiotic at 10 or 30 mg/L in the first treatment batch (Fig 1B and 1C). Thus, our results indicated that the inhibition rate in the second treatment batch was significantly related to the concentration of cefradine in the first treatment batch. However, higher concentrations of the antibiotic did not always cause stronger inhibition. The present results demonstrated that *C*. *pyrenoidosa* exposed to 30 mg/L in the first treatment batch (i.e., Treatment groups 2.1 and 2.2), yielded the strongest inhibition in the second treatment batch for each amount of time. In contrast, the algae that underwent a first treatment batch at 60 mg/L exhibited the minimum inhibition rate in the second treatment batch at the corresponding concentrations and times. Additionally, in the second treatment batch, the inhibition of 60 mg/L cefradine was almost greater than that of 30 mg/L in algae that had treated the same concentration in the first batch. Consequently, these data could be an indication that *C*. *pyrenoidosa* that treated more cefradine in the first treatment batch; obtained a higher level of tolerance to the antibiotic after the treatment; and thereafter could better survive under the impact of antibiotic re-exposure.

**Fig 2:**
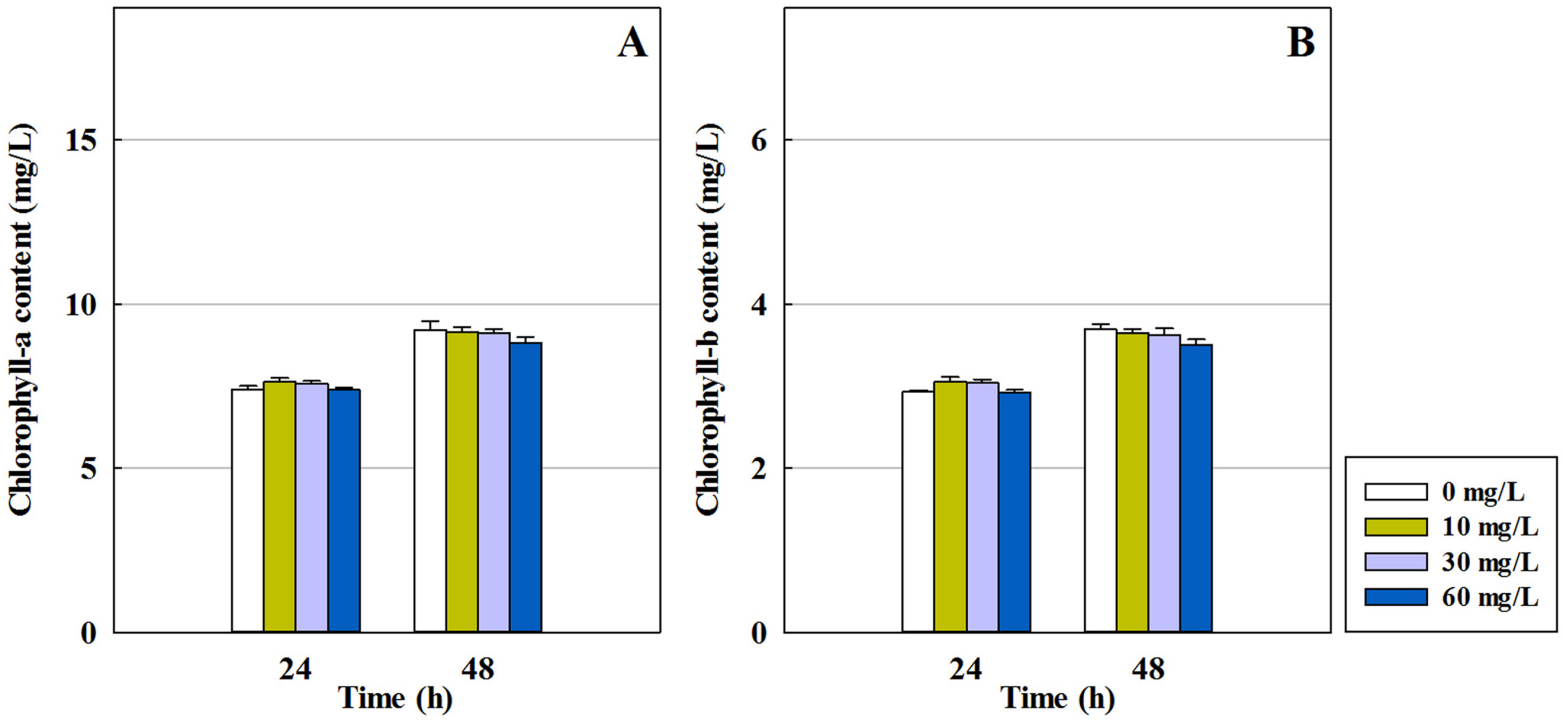
The chlorophyll content of *C*. *pyrenoidosa* in the first treatment batch of cefradine at three concentrations (10, 30 and 60 mg/L). (A) Chlorophyll-a content. (B) Chlorophyll-b content. Algae with under 0 mg/L of the antibiotic were used as the no treatment group.

**Fig 3:**
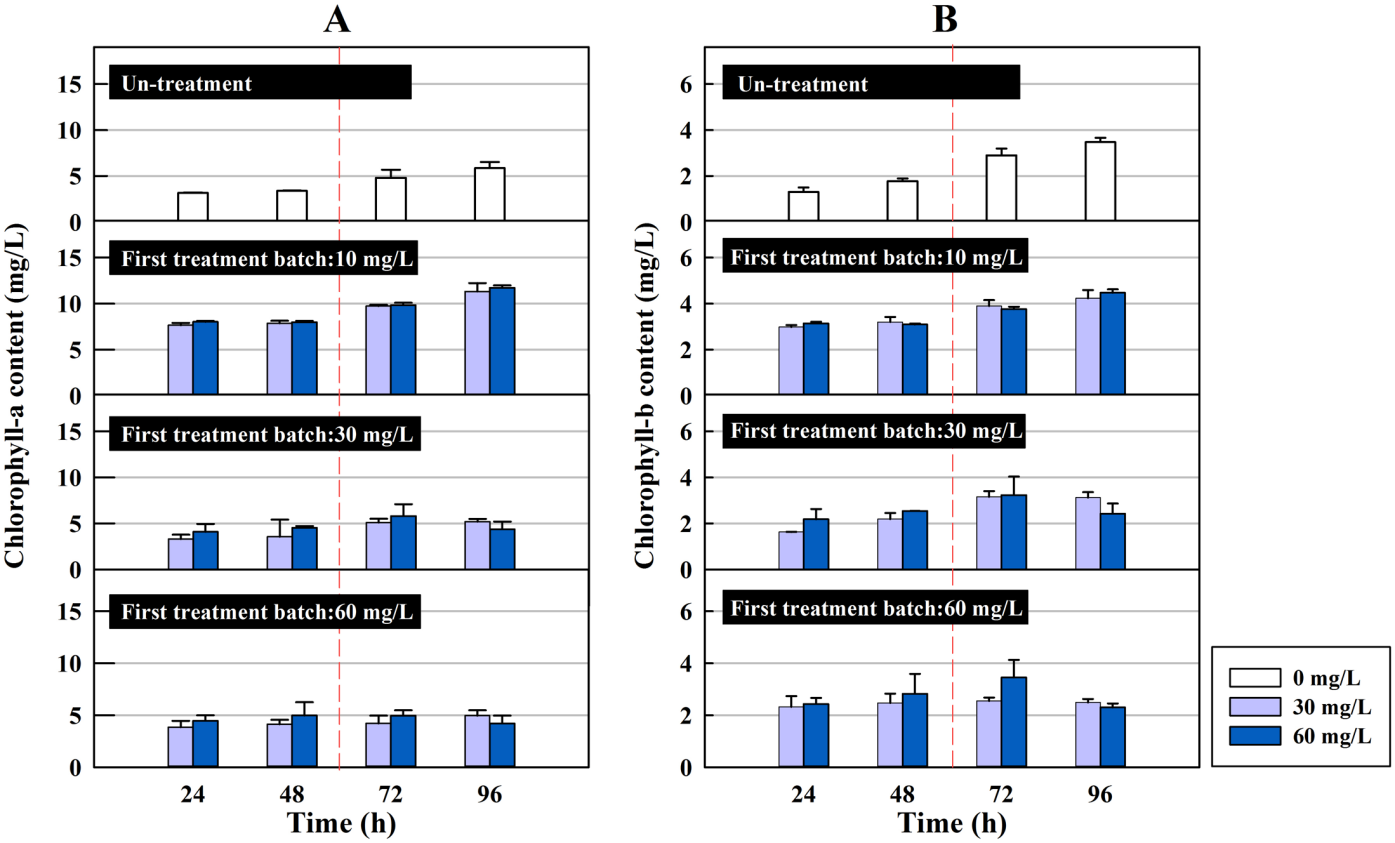
The chlorophyll content of *C*. *pyrenoidosa* in the second treatment batch. (A) The chlorophyll-a content of the algae in the second treatment batch at two concentrations (30 and 60 mg/L) after a first treatment batch at 10, 30 and 60 mg/L. (B) The chlorophyll-b content of the algae in the second treatment batch at two concentrations (30 and 60 mg/L) after a first treatment batch at 10, 30 and 60 mg/L. Algae with 0 mg/L of the antibiotic was used as the no-treatment group.

Chlorophyll is an important pigment for algal photosynthesis and plays a significant role in energy capture and transfer. Chlorophyll biosynthesis in algae is a complicated process that includes sixteen catalytic reactions [28]. In the present study, the chlorophyll-a and chlorophyll-b contents of *C*. *pyrenoidosa* in the first and second treatment batches are shown in Figs 2 and 3, respectively. In the first treatment batch (see Fig 2), there was no a clear difference between the no treatment group and the other groups at the given concentrations of the antibiotic (*p*>0.05). However, in the second treatment batch, cefradine at different concentrations had different effects on the photosynthesis of the algae. This suggests that the impact of various concentrations of the antibiotic on chlorophyll content was stimulated in the second treatment batch.

As shown in Fig 3, the chlorophyll-a and b content of the no-treatment group increased over the course of 48 h, while it was approximately half that when algae were used to treat the antibiotic at 30 and 60 mg/L following the first treatment batch at 10 mg/L (Treatment group 1.1 and 1.2). However, the same trend was not observed in cases in which the first treatment batch had a strength of 30 or 60 mg/L. After the first treatment batch at 30 mg/L, a second treatment at 30 or 60 mg/L resulted in chlorophyll-a and chlorophyll-b content that increased during the first 72 h and then decreased. Similar changes were observed in the second treatment batch at the above two concentrations after a first treatment at 60 mg/L. Therefore, the chlorophyll-a and chlorophyll-b content consistently increased during 72 h in algae that treated higher concentrations in the second treatment batch than in the first one. It is worth noting that at 96 h following the second treatment, the chlorophyll-a and chlorophyll-b content of the algae in Treatment groups 2.1, 2.2, 3.1 and 3.2 was less than that of the no-treatment group, while the algae in Treatment groups 1.1 and 1.2 produced more chlorophyll-a and b. This implied that cefradine at low concentrations stimulated algal photosynthesis. In a previous study, the photosynthesis of *C*. *pyrenoidosa* was always restrained and chlorophyll content decreased with increasing concentrations of bensulfuron-methyl [29]. At the genetic level, the inhibition of chlorophyll by antibiotics was viewed as an interruption of gene expression, which eventually influenced protein synthesis [30]. Furthermore, the impact of antibiotics was structure-dependent. Erythromycin was more strongly inhibitory of chlorophyll biosynthesis in *S*. *capricornutum* than ciprofloxacin and sulflamethoxazole [31].

**Fig 4:**
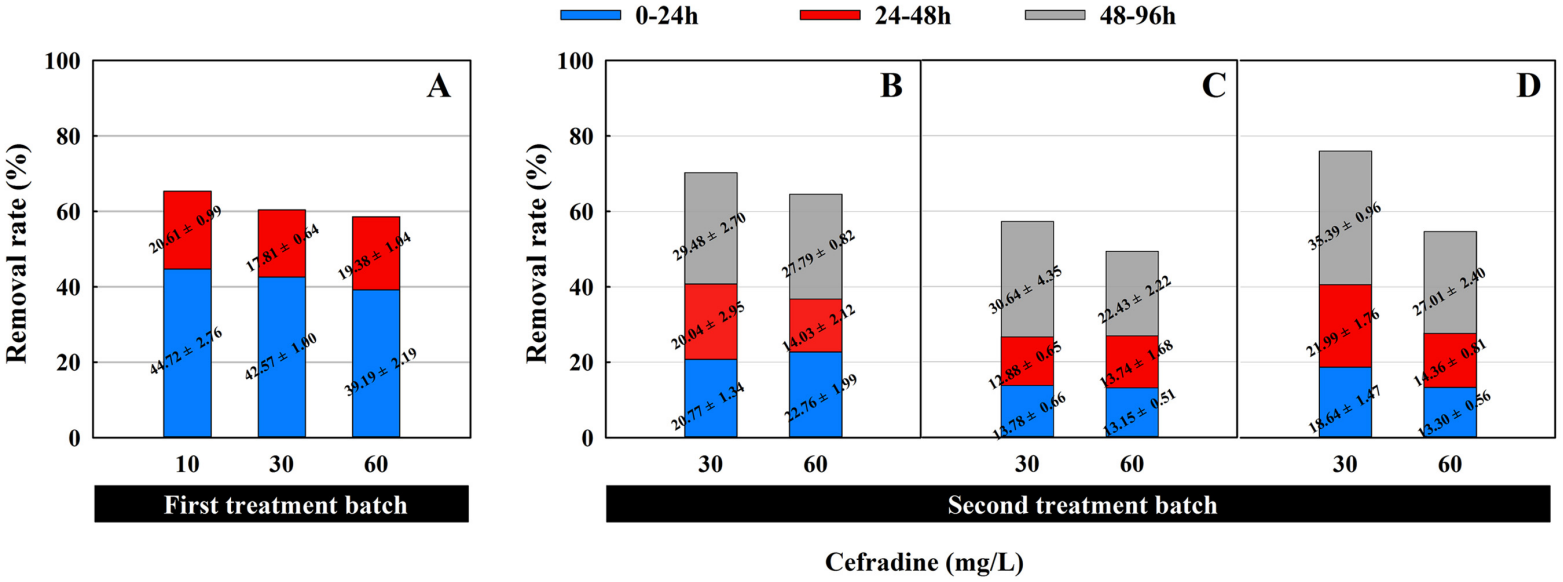
The removal rate of cefradine by *C*. *pyrenoidosa*. (A) In the first treatment batch. (B) In the second treatment after a first treatment batch at 10 mg/L. (C) In the second treatment after a first treatment batch at 30 mg/L. (D) In the second treatment after a first treatment batch at 60 mg/L.

The removal rate of cefradine by green algae in the first and second treatment batches is presented in Fig 4. In the first treatment batch, the removal rate was 44.72 ± 2.76%, 42.57 ± 1.00% and 39.19 ± 2.19% at 10, 30 and 60 mg/L, respectively, during the first 24 h, while the corresponding values during the second 24 h were only 20.61 ± 0.99%, 17.81 ± 0.64% and 19.38 ± 1.04%, respectively (Fig 4A). This suggests that approximately 40.00% of the cefradine was removed in the first 24 h, regardless of the cefradine concentration. However, as shown in Fig 4B, 4C and 4D, when the algae treated cefradine again at 30 mg/L, the removal rate in the first 24 h was 20.77 ± 1.34%, 13.78 ± 0.66% and 18.64 ± 1.47%, respectively. This indicated that the removal capacity of the algae was weaker in the second treatment batch than in the first one. Similar results were observed in the second treatment batch at 60 mg/L. In addition, in the first treatment batch, the removal rates by the algae in the second 24 h were always less than in the first 24 h. Previous studies indicated that algae had a promising and efficient degradation capacity on chemicals. Approximately 99% of the endocrine-disrupting chemical bisphenol A could be removed by the marine microalga *Stephanodiscus hantzschii* in 16 days, while the highest removal rate was observed in 4–8 days [18]. Although more than 66.0% of the herbicide prometryne was degraded after a 5-day treatment with the green alga *Chlamydomonas reinhardtii*, the primary removal process occurred on the last day [20]. In contrast, the present results suggest that the primary reaction of the algae on the antibiotic cefradine occurred in the first 24 h. In the algal treatment process, the algae might bio-accumulated the antibiotic in the cells firstly and then degraded it gradually and slowly. Due to the fast bioaccumulation step, the apparent removal efficiency seemed to be primarily on the first day.

An acceptable removal process not only provides an excellent removal rate but also can adapt to varied pollution loads between treatment batches. The present treatment process provided both of these capacities. On one hand, after a 48 h treatment, the removal rate in the second treatment batch was lower than that in the first treatment batch at any concentration, but a better removal efficiency was obtained when the HRT was extended to 96 h (See Fig 4B, 4C and 4D). The total removal rate of the antibiotic at 30 mg/L in the second treatment batch was 70.29%, 57.30% and 76.02%, respectively, which was 1.16, 0.99 and 1.26 times of that in the first treatment batch. The total removal rate of the antibiotic at 60 mg/L exhibited a similar trend. On the other hand, a comparison of the results in Treatment groups 1.1 and 1.2 suggests that the green algae performed better when they were exposed to a low pollution load (i.e., 10 mg/L) in the first batch and an increased pollution load in the second batch (i.e., 30 or 60 mg/L). We observed 70.29% and 64.59% of cefradine at 30 and 60 mg/L, respectively, was removed in the second treatment batch, which was 1.16 and 1.10 times of that in the first treatment batch at the corresponding concentrations. In addition, microalgae are photosynthetic organisms capable of converting light energy and carbon sources, such as carbon dioxide (CO_2_), into biomass. Unlike activated sludge, the organic content reduction in the input sewage has little or no effect on the green algae. The removal rate under these conditions, which were represented by treatment group 3.1, also indicated that the green algae could adapt to varied pollution loads. Although after the first treatment batch at 60 mg/L, the pollution load was decreased to 30 mg/L, the final removal rate in the second treatment batch increased to 76.02%, which was 1.26 times that in the first treatment batch.

Algal treatment of antibiotics is not a one-way process; the response of the algae to the impact of the antibiotics might influence the removal efficiency in subsequent treatment batches. In general, the sensitivity of algae to antibiotics varies significantly. For instance, the green algae *Pseudokirchneriella subcapitata* was less sensitive than the cyanobacterium *Anabaena* CPB4337 in response to levofloxacin and norfloxacin, while tetracycline was more toxic to green algae [32]. Moreover, 96 h-EC_50_ values of toxic effects of chloramphenicol, florfenicol and thiamphemicol on the freshwater algae *C*. *pyrenoidosa* were 14.0, 215.0 and 1283.0 mg/L, respectively, which ranged across more than one order of magnitude [33]. The present study also demonstrated the impact of cefradine on the growth of *C*. *pyrenoidosa*. Our previous study demonstrated that the population growth of *Microcystis aeruginosa* was inhibited significantly when exposed to cefradine for 6 days [34]. The population of *M*. *aeruginosa* declined continuously at 5 and 12 mg/L of cefradine, while the chlorophyceae *Scenedesmus obliquus* was able to grow when used to treat the antibiotic. Compared with the algae in the first treatment batch, the “impact background” of the algae in the second treatment batch warrants attention. Although a previous study demonstrated that algae were able to bioaccumulate and degrade pesticides and thereafter improve toxic tolerance [35], higher tolerances might sometimes be viewed as a response of an organism to the pollutant. Algal re-exposure to pesticides was not reported in the above work. Similar research on antibiotics has also been limited. In addition, toxic stress on the organism might also persist even if the exposure was ended. Previous research also indicated the pesticide dimethoate depressed the feeding behavior of rotifers at 8 h post exposure [36]. Thus, toxic stress might persist even after the first treatment batch ends, and the results from the second treatment batches in the present study were highly process-dependent. Variable responses in our treatment included the post-exposure effect at different times following first exposure, the response of the algae to the second treatment batch, and the response to varying antibiotic concentrations; all of these factors may causally influence the process. Thus, there were not only effects of the concentrations of the individual antibiotics given in the first treatment batch but also combined effects, which included post-treatment toxicity of the antibiotics after the first treatment batch and the impact of the antibiotic on the second treatment batch. In addition, after the fast bio-accumulate step, the primary degradation process should happen later. It is possible that the bio-accumulate step in the second batch might occurred with the degradation process of the first batch synchronously. Thus, the difference of the concentration between the two batches might influence the alga and the treatment process. Our present work indicates that the impact of cefradine on *C*. *pyrenoidosa* and the algal removal capacity with respect to the antibiotic varied. To the best of our knowledge, this study is the first to consider the toxic background and feedbacks present during different treatment batches. The algal metabolite in the two treatment batches should be considered in our future study, which should help to explain clearly about how the algae to remove antibiotics.

**Fig 5:**
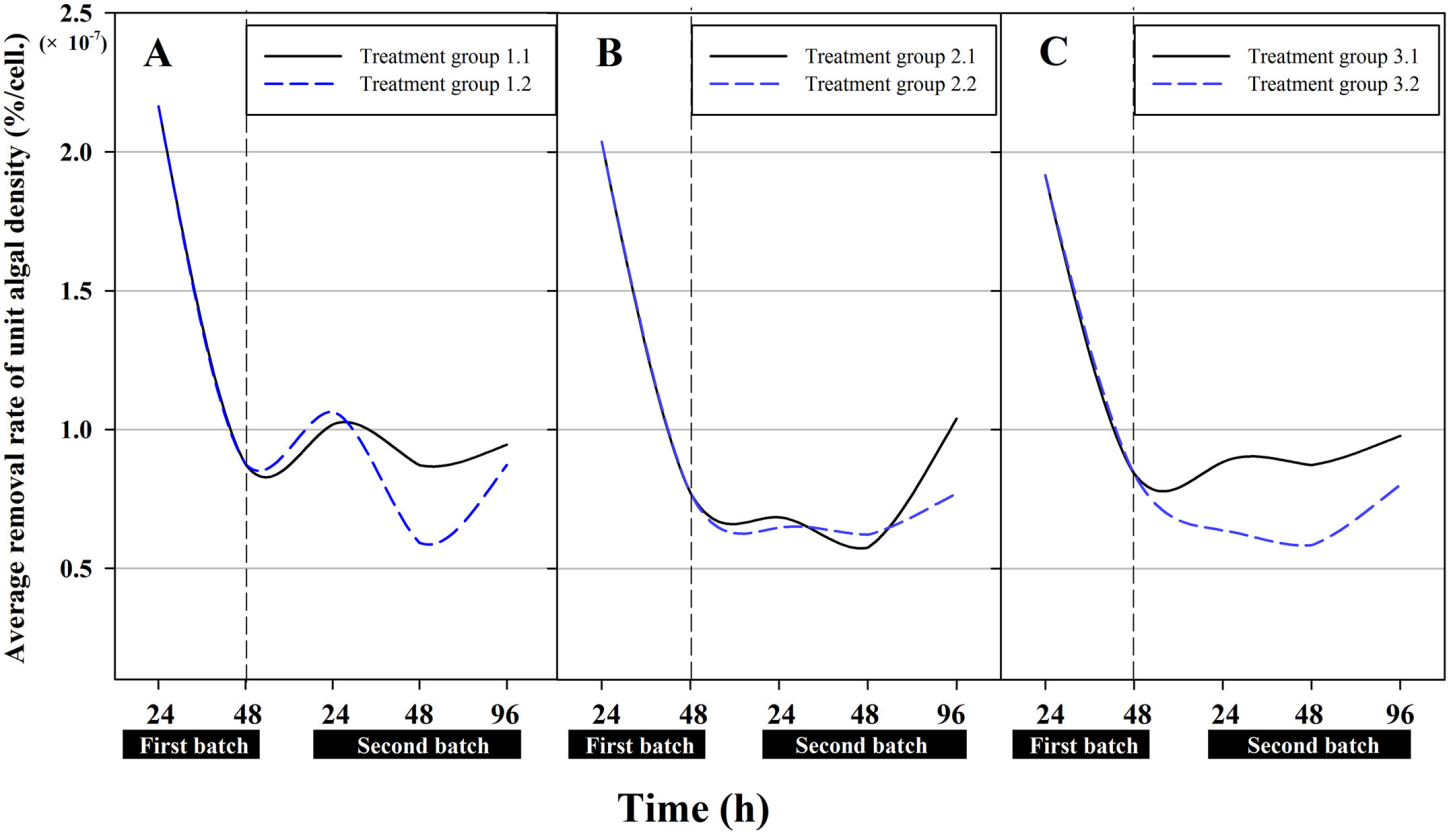
The removal rate of cefradine by a unit algal cell. (A) in Treatment group 1.1 and 1.2. (B) in Treatment group 2.1 and 2.2 (C) in Treatment group 3.1 and 3.2.

It should also be noted that all removal rates in previous studies were reported for the algal population as a whole. Considering that algal population density also changes during the removal process, the removal rate of the unit algal density per hour, i.e., the “cellular removal rate”, should better reflect the removal capacity at any given time. As shown in Fig 5, after a first treatment at 10, 30 and 60 mg/L, the removal rate of the unit algal density varied when the algae treated the antibiotic at two different concentrations in the second batch. If the algae treated cefradine at the lowest concentration (10 mg/L) in the first batch, the removal rate of the unit algal density improved during the first 24 h and thereafter fell slightly up to 48 h in the second batch at 30 and 60 mg/L. However, a similar change was observed in the second treatment batch at 60 mg/L no matter the alga treated the antibiotic at 30 or 60 mg/L in the first treatment batch (Fig 5B, Treatment group 2.2, and Fig 5C, Treatment group 3.2). In addition, an improved removal rate per algal cell was obtained when the alga treated the antibiotic at 30 or 60 mg/L in the first treatment batch, respectively and at 30 mg/L in the second treatment batch (Fig 5B, Treatment group 2.1, and Fig 5C, Treatment group 3.1).

The green algae in the present study had a high total removal efficiency under the impact of cefradine, even at higher concentrations, while algal population growth was inhibited continuously. This suggests that the algal cells in the second treatment batch retained their ability to remove cefradine; nonetheless, because of the impact on the algae from the first treatment batch, the cellular removal capacity of the antibiotic slowed down in the second treatment batch. However, compared with the no-treatment algae, the chlorophyll-a content increased during the second treatment, as did the chlorophyll-b content. One reason for this was most likely that the toxicity of cefradine and its degradation products increased gradually. Therefore, cefradine in the first treatment batch had a relatively weak toxic effect on the photosynthetic system, so *C*. *pyrenoidosa* was able to recover quickly and promote chlorophyll-a biosynthesis in the second treatment batch. These observations suggest that algae might produce more photosynthetic pigments as a passive response to the impact of antibiotics. From a nutritional point of view, microalgae are predominantly photoautotrophic but they can also be heterotrophic, a lifestyle characterized by the consumption of organic carbon as a carbon source. The present study suggests that green algae increased their photosynthetic capacity in response to the impact of cefradine; additionally, the degraded antibiotic might serve as an organic carbon source.

## Conclusion

During the first treatment batch, the antibiotic cefradine influenced the biomass of the green algae *C*. *pyrenoidosa*. Meanwhile, the “toxic background” of the algae also produced a physiological response and degraded the antibiotic in the subsequent treatment batch. Although the maximum population inhibition rate was observed 96 h after the second treatment batch for all concentrations (see Fig 1B, 1C and 1D), the removal rate of the unit algal density reached its peak at varying concentrations (see Fig 5). Light is essential for the metabolism of photosynthetic microalgae. Due to the wide application of algae to remove environmental contaminants, a question arises regarding whether the removal rate for subsequent treatment batches will be improved if the algae are allowed to recover under abundant and artificially intensified light conditions after the first treatment batch; this topic is worthy of further research.
